# Preparation and effects of nano mineral particle feeding in livestock: A review

**DOI:** 10.14202/vetworld.2015.888-891

**Published:** 2015-07-21

**Authors:** Partha Sarathi Swain, D. Rajendran, S. B. N. Rao, George Dominic

**Affiliations:** 1 Division of Dairy Cattle Nutrition, National Dairy Research Institute, Karnal - 132 001, Haryana, India; 2 Animal Nutrition Division, National Institute of Animal Nutrition and Physiology, Bengaluru - 560 030, Karnataka, India

**Keywords:** biological effects, mineral nutrition, nanotechnology, nano Zn, synthesis

## Abstract

Nano minerals are widely used in diversified sectors including agriculture, animal, and food systems. Hence, their multiple uses provoke the production of nanomaterials at the laboratory level, which can be achieved through physical, chemical or biological methods. Every method is having its own merits and demerits. But keeping all in mind, chemical methods are more beneficial, as uniform nano-sized particles can be produced, but the use of corrosive chemicals is the main demerits. When it comes to environmental issues, biological methods are better as these are free from corrosive chemicals, but maintaining the culture media is the disadvantage. For animal feeding, chemical methods are mostly followed to produce nano minerals as it is cheap and less time consuming. These nano minerals also showed their significant effects even at lower doses of recommendations than the conventional mineral sources. These nano minerals have significant growth promoting, immuno-modulatory, antibacterial effects than the conventional counterparts. They also alter the rumen fermentation pattern on supplementation in the animal feeds. Apart from these, nano minerals are reported to enhance the reproduction in the livestock and poultry.

## Introduction

This era may be conferred as the era of nanotechnology due to the use of nanoparticles in diversified purposes such as in the fields of medicine, engineering, information, environmental technology [1,2], pigments, food, electronics appliances [3], biological, and pharmaceutical applications [4,5], etc. Nanomaterials are also used in the fields of biology (molecular and cellular), biotechnology, mineral nutrition, physiology, reproduction, pharmacology, etc., in both animal and human models. Hence, nanotechnology and in turn, the nano-sized materials are having multifaceted use in agriculture and food systems. The applications of nanomaterials in agriculture and animal husbandry are of utmost importance since the Indian economy is predominantly depending on agriculture [6]. Nanotechnology is concerned with materials whose structures exhibit significantly novel and improved physical, chemical, and biological properties due to their nano-scaled particle size [7]. This can be defined as a research and development aimed at understanding and working with seeing, measuring, and manipulating matter at the atomic, molecular, and supramolecular levels [8].

National Nanotechnology Initiative (NNI) defined Nanotechnology as “utilization of structure with at least one dimension of nanometer (nm) size for the construction of materials, devices or systems with novel or significantly improved properties due to their nanosize” [9]. In simple terms, nano mineral particles refer to the particle having a particle size in the range of 1-100 nm [10-12]. At this scale, the physical, chemical, and biological properties of materials differ fundamentally and often unexpectedly [11]. The nano-sized particles are having higher potential than their conventional sources and thus reduce the quantity required [6].

The aim of the present review was to put light on different properties of nano-sized materials along with the methods to prepare them. Along with this, we will review their biological effects on livestock.

## Properties of Nano Minerals

Nano minerals are essentially having a particle size of 1-100 nm. These are stable under high temperature and pressure [4] and can be easily taken up by the gastrointestinal tract and utilized in the animal system, so are more effective than the larger sized ZnO at lower doses [12]. It results in better interaction with other organic and inorganic substances due to its more surface area *in vivo* [13]. Nanosized ZnO also has minimal adverse effect on human cells [14] which is an added advantage. Nano minerals can cross the small intestine and further distribute into the blood, brain, lung, heart, kidney, spleen, liver, intestine, and stomach [15]. The intrinsic properties of nanometals are mainly determined by its size, shape, composition, crystalline structure, and morphology [16]. The functional activities such as chemical, catalytic or biological effects of nano minerals are heavily influenced by their particle size [17,18].

## Effect of Nano- mineral Feeding on Livestock Production

This nanoparticle has produced positive responses when fed as an alternative to the conventional mineral sources. If we talk about the Zn as an example, feeding nano Zn to the livestock and poultry has produced encouraging responses in growth, immunity, and reproduction. Nano ZnO is found to enhance growth, improve the feed efficiency in piglets [19], and poultry [20,21]. Nano Zn improves the immunity of the animals. For an instance, a reduction in somatic cell count in subclinical mastitis cow and an increase in milk production was observed due to supplementation of nano ZnO [22]. Yang and Sun [19] also reported a significant drop in the diarrhea incidences by supplementing graded doses of nano ZnO in the diet. Sahoo *et al.*, [23,24] observed an improved immunity of the birds by supplementing nano Zn at 0.06 ppm to the basal diet of broilers in comparison to conventional dose of 15 ppm of organic and inorganic Zn. Nano Zn also changes the rumen fermentation kinetics in ruminants and also found to alter the proportion of the volatile fatty acids produced. Supplementation of nano ZnO, *in vitro*, showed an improvement in the growth of ruminal microorganisms, ruminal microbial protein synthesis, and energy utilization efficiency in the early phase of incubation [25]. Nanosensors are available to study the causes of abortion. Nano antioxidant can prevent retained placenta and other reproductive problems after calving and for improving infertility problems. New application in animal production system is nanotube implanted under the skin to provide real-time measurement of the level of estradiol in the blood [22]. Thus, supplementation of nano Zn to animals can possibly eliminate these reproductive disturbances and thus improve the economics of farming.

## Methods to Prepare Nano Particles

This multifocal use of nanomaterials has created a huge demand, which leads to develop some effective and sensitive methods to synthesize desired nanoparticles. The prime aim of synthesizing these nano minerals is to have a better control over particle size, morphology, purity, quantity, and quality [26]. Nano minerals can be synthesized by physical, chemical, and biological methods. In general, biological methods are safe to use and can be efficiently exploited without further experiment on the residual effect [22] and are eco-friendly too [6]. For animal feeding, nanoparticles can be produced by any of the above methods but thorough study including toxicological effect is advocated before using these particles in the ration of livestock and poultry on a routine basis.

### Physical methods

Different researchers have mentioned a wide range of the physical methods for preparation of nanoparticles like, evaporation-condensation, which might be carried out using a tube furnace at atmospheric pressure [27], evaporation-condensation and laser ablation together [28], electric arc discharge, chemical vapor deposition, gas phase synthesis and ball milling-annealing methods [29], physical vapor deposition [30], etc. Ball mill is used to grind materials into extremely fine powder of nano size for use in livestock feeding [31-33]. High energy ball milling (HEBM) can be used for synthesizing the nano minerals, which can provide 1000 times high impact grinding than traditional ball mills. However, a longer milling time is generally required for HEBM to activate and complete the structural changes. While using the HEBM, controlled milling atmosphere and temperature are to be taken care carefully. The demerit in the gas phase synthesis of nanomaterials is that it usually results in deposits of particles with larger size distributions, in some cases ranging from 10 nm to 200 nm [29]. The absence of solvent contamination and maximum recovery of nano minerals are the advantages of physical methods of nano mineral synthesis [28].

### Chemical methods

The chemical methods are having an upper hand over physical method with respect to the stabilization of nano mineral particles from agglomeration, extraction of nano mineral particles from solvent, surface modification, processing control, and mass production [29]. In the physical method, the produced nanoparticles are having a wider range of particle size. But in chemical method, a uniform nano-sized particle can be produced [34]. Hence, effective and controlled bulk production can be achieved by using the chemical methods. Reduction of mineral salts by chemical methods is the most convenient way to reduce the size of the particles [29]. However, in chemical method there is always a chance of toxicity as hazardous chemicals are used during synthesis. Hence, attempts are made to produce nano mineral particles by using eco-friendly chemicals, plant and fungal origin as reducing agents [35], called as green chemistry method of nanoparticle synthesis. Eco-friendly as well as nontoxic chemicals such as glucose, starch, amino acids, and plant extracts are used to synthesize metal nano mineral particles. Alternatively, sonochemical method and microwave synthesis are the alternatives to toxic chemicals methods for synthesis of nano mineral particles in large scale and in cost effective manner as well [29]. The particle size of the minerals depends largely on the reduction capability of the reagents, for an instance, a strong reducing reagent promotes a fast reaction rate and produces smaller nano mineral particles [29]. In the chemical method of nanoparticle synthesis, stabilizing agents and surfactants such as cyclodextrin, polyvinyl pyrrolidone, polyvinyl alcohol, citrate or quaternary ammonium salts, etc., are needed to prevent the agglomeration of the metal particles [36]. Solvent molecules can stabilize the nano mineral particles more efficiently [37]. The stabilizers prevent the uncontrollable particle size reduction, check the produced nano minerals particle aggregation, control of particle size, and also allow particle solubility in various solvents [38]. Ligands such as phosphines, thiols, amines, carbon monoxide, etc., can also be used as a stabilizer in nano mineral particle production, which occurs through coordination between the ligand moiety and metal nano mineral particles [29].

### Biological methods

The conventional methods are usually hazardous and energy consuming. This leads to focus on “green synthesis” of nano mineral particles which seems to be an easy, efficient, and eco-friendly approach [39] and also minimize the toxicity [40]. Biosynthetic methods using either biological microorganisms or plant extracts have emerged as a simple alternative to physical and chemical methods [6]. Over the past several years, plants, algae, fungi, bacteria, and viruses have been used for the production of low cost, energy-efficient, and non-toxic nano mineral particles [6,35,41]. Various metal nanoparticles such as silver, gold, cadmium, selenium, palladium, barium titanate, and titanium has been successfully synthesized by biological methods [40,42-44] by using different plant materials but the biosynthesis of ZnO nanoparticle is in infancy. Sri Sindhura *et al.*, [6] prepared nano Zn by using leaves of *Parthenium hysterophorous*.

Use of plant materials for the synthesis of nano minerals is easier and advantageous as this method is simple and is having upper hand as it follows single step synthesis procedures, one pot synthesis, easy product recovery from the final solutions, eco-friendly, compatible to pharmaceutical and biomedical applications, cost effective, economic viability, nontoxic, less time consuming, and no need to maintain selected culture [29]. Metal nano mineral particles have been successfully synthesized from *Avena sativa, Azadirachta indica, Aloe vera*, alfalfa, lemongrass, *Sesbania drummondii*, papaya fruit extract, and latex of *Jatropha cutcas* [45]. In spite of having so many advantages, maintaining the culture and its condition, culture media, time period in formation of the nano mineral particles, and difficulty in product recovery are among the main drawbacks of biological method of nano mineral particles synthesis.

## Conclusions

Nano minerals are having a great potential as mineral feed supplements in animals even at very lower doses than the conventional organic and inorganic sources. However, the systematic and thorough studies are to be undertaken to see the toxic effects if any after feeding animals for prolong period. Again, these nano mineral particles can be synthesized in the laboratory by using simple instruments and chemicals. The economics and stability of the produced nano mineral particles under normal storage condition are to be studied. Among the different preparation methods, the chemical method is the easiest and most economical as only few chemicals can produce the nano mineral of uniform size in the laboratory. However, proper precaution while using the chemicals should be followed. Moreover, these synthesized mineral nanoparticle should be fed to a large number of animals to standardize both the positive and adverse effects before incorporating in the ration on a regular basis.

## Authors’ Contributions

PSS is a Ph.D. scholar from National Dairy Research Institute, Karnal under the major supervision of SBNR. SBNR designed the review and PSS collected the literatures and drafted the manuscript. DR and GD were involved in critically evaluating the manuscript and provided the guidance. All authors read and approved the final manuscript.
